# Genes of Both Parental Origins Are Differentially Involved in Early Embryogenesis of a Tobacco Interspecies Hybrid

**DOI:** 10.1371/journal.pone.0023153

**Published:** 2011-08-04

**Authors:** Jun-E Zhang, An Luo, Hai-Ping Xin, Jing Zhao, Shi-Sheng Li, Liang-Huan Qu, Li-Gang Ma, Stefan Scholten, Meng-Xiang Sun

**Affiliations:** 1 Key Laboratory of Ministry of Education for Plant Development Biology, College of Life Science, Wuhan University, Wuhan, China; 2 College of Life Science, Jiangxi Normal University, Nanchang, China; 3 Biocenter Klein Flottbek, Developmental Biology and Biotechnology, University of Hamburg, Hamburg, Germany; Temasek Life Sciences Laboratory, Singapore

## Abstract

**Background:**

In animals, early embryonic development is largely dependent on maternal transcripts synthesized during gametogenesis. However, in higher plants, the extent of maternal control over zygote development and early embryogenesis is not fully understood yet. Nothing is known about the activity of the parental genomes during seed formation of interspecies hybrids.

**Methodology/Principal Findings:**

Here, we report that an interspecies hybridization system between SR1 (*Nicotiana tabacum*) and Hamayan (*N. rustica*) has been successfully established. Based on the system we selected 58 genes that have polymorphic sites between SR1 and Hamayan, and analyzed the allele-specific expression of 28 genes in their hybrid zygotes (Hamayan x SR1). Finally the allele-specific expressions of 8 genes in hybrid zygotes were repeatedly confirmed. Among them, 4 genes were of paternal origin, 1 gene was of maternal origin and 3 genes were of biparental origin. These results revealed obvious biparental involvement and differentially contribution of parental-origin genes to zygote development in the interspecies hybrid. We further detected the expression pattern of the genes at 8-celled embryo stage found that the involvement of the parental-origin genes may change at different stages of embryogenesis.

**Conclusions/Significance:**

We reveal that genes of both parental origins are differentially involved in early embryogenesis of a tobacco interspecies hybrid and functions in a developmental stage-dependent manner. This finding may open a window to seek for the possible molecular mechanism of hybrid vigor.

## Introduction

During fertilization, a sperm cell fuses with an egg cell and thus paternal and maternal genomes carrying genetic information combine together. During zygotic development or early embryogenesis a maternal-to-zygotic transition occurs, which depict the time point when the embryonic genome take over control of its own development. In animals, e.g. mammals, there is a delay between fertilization and the maternal-to-zygotic transition and thus early embryogenesis is largely dependent on maternal transcripts deposited in the egg cell before fertilization. The length of this delay is species dependent. Activation of the embryonic genome occurs at 2-celled stage in mice, the 28-celled stage in C. elegans, and at the mid-blastula stage in Xenopus [1]–[4]. As a result, early events involved in cell division or pattern formation are programmed almost entirely though maternal mRNA.

However, the parental origin of genes involved in early embryogenesis of higher plants is still largely unknown. It was reported that the activity of many genes are largely transcribed from the maternally inherited alleles and transcriptional activation of paternal genome during early development is delayed [5]–[7], leading to the conclusion that maternal transcripts might be sufficient to direct early embryogenesis. Paternal silencing or at least a strong reduction in the expression of paternal alleles has also been independently confirmed for several developmental genes or markers [6], [8]. Newly reported data of DNA polymerase inhibition by RNAi after fertilization suggest that maternal transcripts are sufficient for early embryogenesis [9]. At the same time, some evidences have been presented for early-transcribed paternal alleles [10]–[12]. Moreover, Bayer (2009) showed paternal control of embryonic patterning in *Arabidopsis thaliana*. Thus, it seems that paternal transcripts are instrumental in regulating important aspects of plant zygote development and early embryogenesis [13].

Recently, Meyer and Scholten (2007) evaluated for the first time the relative levels of parental transcripts in zygotes and provided evidence for equivalent parental contribution to early plant zygotic development in maize. They found an extensive activation of the paternal genome is consonant with observations of high levels of heterosis in early hybrid maize embryos [14].

To answer the question if genes of both parental origins also equivalently contribute to interspecific hybrids during early embryogenesis, we established tobacco as an experimental system due to availability of relevant techniques for the isolation of zygotes and proembryos. We first selected a large number of ESTs of tobacco from NCBI (http://www.ncbi.nlm.nih.gov/) to detect polymorphism between two tobacco species, SR1 and Hamayan by SSR (Single Sequence Repeats). We established a cross system for interspecies hybrid (Hamayan × SR1) and tested the parental origin of the ESTs that were expressed in both parents and the hybrid at the same developmental stage by PAGE (SDS polyacrylamide gel electrophoresis). The result confirmed the involvement of paternal transcripts in early embryogenesis of tobacco, and revealed differential contribution of the parental genomes during early hybrid embryogenesis.

## Results

### Establishment of an interspecies cross system in tobacco (Hamayan × SR1)

**Figure 1:**
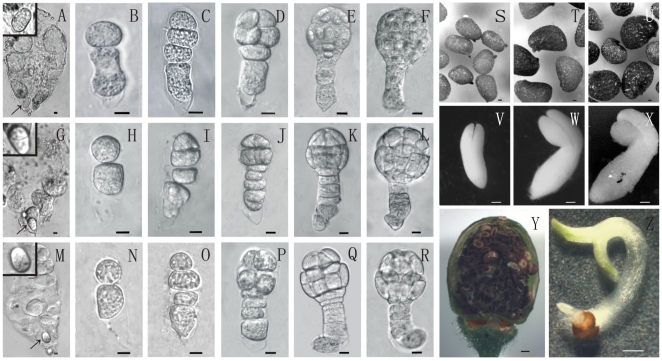
Early Embryogenesis, Mature Embryos and Ovules in Tobacco SR1, Hamayan and Their Hybrid (Hamayan × SR1). A-F: Isolated zygote embryo sac and proembryos at different stages of SR1. G–L: Isolated zygote embryo sac and proembryos at different stages of the hybrid. M–R: Isolated zygote embryo sac and proembryos at different stages of Hamayan. Bar = 10 µm; arrows indicate zygotes in embryo sacs as shown in the insertions. (S). Normal ovules of SR1. (T) Normal ovules of Hamayan; (U). Normal ovules of hybrid; (V). Mature embryos of SR1; (W). Mature embryos of Hamayan; (X). Mature hybrid embryos. Bar = 0.1 mm; (Y). Sterile ovules in a hybrid ovary; (Z). A isolated normal hybrid embryo that developed in vitro. Bar = 0.5 mm.

During pre-experiments, two tobacco species, *Nicotiana tabacum* var. SR1 and *N. rustica* var. Hamayan, were selected for their successful interspecies hybridization. To ensure normal hybrid embryogenesis, we carefully observed early embryonic development both in the controls and the hybrid. The results showed that the cell morphological development of hybrid embryos were normal in vivo (Figure 1). Generally, the cell division pattern and morphology of hybrid embryos look similar to parental embryos, which were used as controls.

The developmental time course of early embryogenesis in SR1, Hamayan, and the hybrid was also analyzed. The results showed that the beginning time of embryogenesis in the three kinds of ovules was different (Figure. S1). The division of the zygote occurred around 100HAP in SR1, whereas it occurred around 40HAP in Hamayan, and in Hamayan × SR1. At the globular embryo stage, hybrid embryos became gradually aborted and about 2% (n = 1599) ovules contained normal embryos at 13–15 DAP. Such normal hybrid embryos were usually bigger than their counterparts in both parental species (Figure. 1).

Embryo rescue technique was used to rescue hybrid embryos. Hybrid ovules were dissected from ovaries at 5–7 DAP for culture in vitro [15]. The results showed that hybrid embryos could develop into normal mature embryos (Figure. 1) and the successful rate is 15% (n = 301). This indicates that endosperm disfunction might be the cause of embryo abortion mentioned above.

The reciprocal hybrid (SR1 × Hamayan) was also performed and analyzed. The size of the pollinated ovaries and ovules at 96HAP were similar to that of SR1 without pollination and much smaller than that of SR1 with self pollination at the same time (Figure S2), indicating the hybridization was not successful. Further investigations revealed that the reason for the failed cross is that the style of SR1 is obviously longer than that of Hamayan (Figure S3). When SR1 stigmas were pollinated with Hamayan pollen, the pollen germinated well, but the pollen tubes could not grow long enough to reach the SR1 embryo sacs (Figure S4). By cutting the SR1 ovaries and putting Hamayan pollens directly on the cutting end, the fertilization was partially successful and early embryos were observed (Figure S5; Table S1). This indicates that the fertilization and the early embryogenesis were at least compatible between the two species. However, due to the low frequency of successful fertilization, no enough synchronously developed hybrid embryos could be obtained for the detection of parental-origin gene expression. Therefore, in the present work the cross system (Hamayan × SR1) was mainly used to analyze the parental-origin transcripts during early embryogenesis in tobacco interspecies hybrids.

### Construction of cDNA pools of sperm cells, zygotes and 8-celled embryos

**Figure 2:**
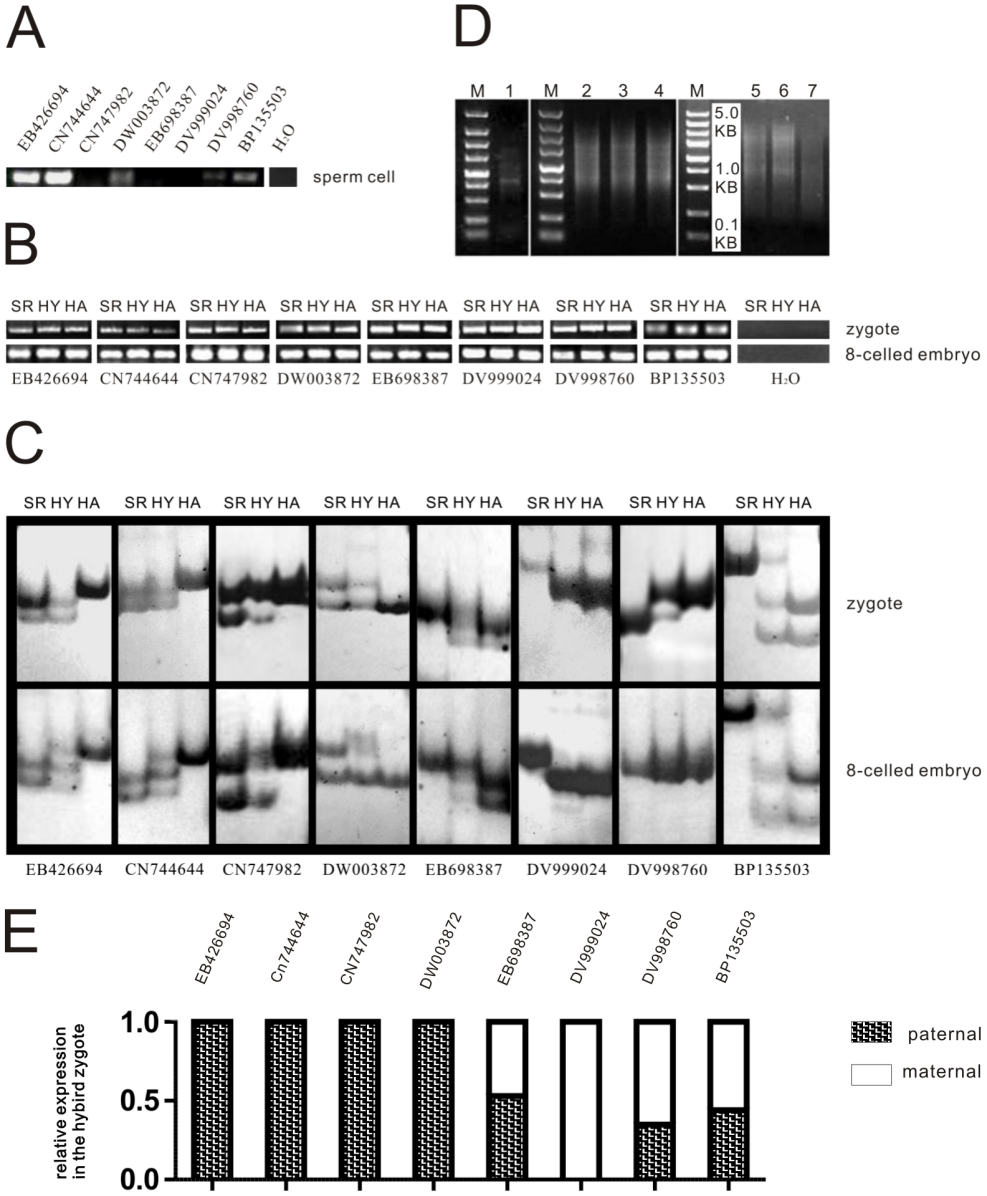
The Expression Detection of 8 Genes at Sperm Cell, Zygote, 8-Celled Embryo Stages and cDNA Pools of SR1, Hamayan and Their Hybrid (Hamayan × SR1). (A) and (B). PCR result. Eight genes were detected in sperm, zygote and 8-celled embryo cDNA pools; (C). PAGE result; SR: SR1; HY: hybrid (Hamayan × SR1); HA: Hamayan. the parental-origin of each gene was directly analyzed by comparing the PCR products from the three different cDNA templates (SR1, hybrid and Hamayan). The PCR products were separated by PAGE. (D). 1: cDNA pool of sperm cell from SR1; 2–4: cDNA pools of zygote from SR1, Hybrid and Hamayan, respectively; 5–7: cDNA pools of 8-celled embryo from SR1, Hybrid and Hamayan, respectively; M: marker. (E). The relative level of maternal and paternal transcripts in hybrid zygote according to the PAGE results.

A well established method based on enzymatic maceration combined with brief dissection [16]–[18] allowed us to isolate enough living sperm cells, zygotes and 8-celled embryos for the construction of cDNA pools. Living sperm cells were obtained from SR1 and living zygotes, 8-cell embryos were obtained from the SR1,Hamayan and their hybrid (Hamayan × SR1) respectively. Their mRNA was isolated, and cDNA was synthesized and amplified. Each individual cDNA pool was then successfully constructed (Figure 2).

### Polymorphic ESTs selection and the expression pattern detection at zygote and 8-celled embryo stages

For there are abundant SSR polymorphism in tobacco, ESTs of tobacco were searched through NCBI. A DNA sequence assembly program CAP3 was used for the elimination of redundant sequences. Then Loci were checked by the MISA(MIcroSAtellites identification tool)for the identification and location of the SSR sites.

Finally, 28 genes were found to express in hybrid zygotes and 8-celled embryos (Hamayan × SR1) (Table 1). In succession, The 28 genes were checked in zygote and 8-celled embryo cDNA pools of SR1 and Hamayan respectively. The PCR results of 8 genes that had confirmable parental origins were displayed. The result showed that all of them were expressed in the parental cDNA pools of both zygote and 8-celled embryo (Figure 2).

**Table 1:** BlastX Sequence Similarity of the Genes Expressed in the Hybrid Zygotes.

Accession	BlastX Sequence Similarity a (Accession Number)	E Value
**EB426694**	**None**	**-**
**CN744644**	**None**	**-**
**CN747982**	**Nucleosome assembly protein 1-like protein 3 [Nicotiana tabacum] (CAD27462)**	**5e-12**
**DW003872**	**None**	**-**
**DV999024**	**SAUR family protein [Populus trichocarpa] (XP_002321970)**	**8e-31**
**EB698387**	**None**	**-**
**DV998760**	**F-box family protein [Citrus trifoliata] (ACL51019)**	**5e-89**
**BP135503**	**Protein phosphatase 2C family protein / PP2C family protein [Arabidopsis thaliana] (NP_187868)**	**5e-20**

a Maximum similarity is obtained using BlastX against the NCBI's nr database.

### Parental-origin specific detection of eight genes in hybrid zygotes and 8-celled embryos

At zygote stage the parental-origin of each gene was directly analysed by comparing the PCR products of reactions from the three different cDNA templates SR1, hybrid and Hamayan. The PCR products were separated by PAGE. This analysis showed that among the eight genes expressed in the hybrid zygote, EB426694, CN744644, CN747982 and DW003872 are of paternal-origin; whereas DV999024 were of maternal-origin, and EB698387, DV998760 with BP135503 were of biparental-origin. This result confirmed that paternal genes was involved in the early embryonic development of interspecies hybrid and also confirmed that the alleles from biparents differentially contributed in the development of interspecies hybrid zygote.

Expression dynamics of each gene was also detected in 8-celled embryo. CN747982, DW003872, EB698387, DV999024, DV998760 and BP135503 expressed as they did in zygote, but EB426694 and CN744644 turned to be biparental-origin, while their maternal transcripts didn't appear in zygote. The changes obviously showed that paternal and maternal alleles differently involved in embryogenesis at the different developmental stage (Figure 2). These data indicate that the expression pattern of allele specific genes during early embryogenesis is developmental stage-dependent.

### The expression detection of eight genes in sperm cells

To reveal if the paternal transcripts in zygotes were inherited from the sperm cells or are the result of *de novo* transcription in zygote, a cDNA pool of sperm cell from SR1 was constructed. The results revealed that in sperm cells EB426694 and CN744644 were strongly expressed, and DW003872, DV998760 and BP135503 were weakly expressed. Notably, CN747982, EB698387 and DV999024 did not show any expression in sperm cells. Thus, the paternal transcripts of CN747982 and EB698387 in zygotes was the result of paternal *de novo* transcription after fertilization. Thus, the paternal transcripts of these genes in zygotes may come from either inheritance or de novo transcription (Figure 2).

## Discussion

### Paternal transcript involvement in hybrid zygote development

The involvement of paternal transcripts in zygote development and early embryogenesis has long been discussed. Pioneer works offered different opinions. Early reports indicated maternal information deposited in egg cells could support embryo development until the proembryo stage and thus the authors tended to the view that early embryogenesis is predominantly maternal-controlled [5], [7]. On the other hand, paternal transcripts were found to be expressed during early embryogenesis. Weijers (2001) revealed detectable paternal gene expression in most embryos of *Arabidopsis* as early as the two-celled stage by strong AtRPS5A::GUS expression [10]. An investigation of the timing of transgene activation after fertilization even showed *de novo* GUS activity already in *Arabidopsis* zygotes [8]. In maize, a transgene driven by a 35S promoter in the paternal genome was almost immediately transcribed in the zygote after fertilization [11]. Our previous work also confirmed that paternal transcripts in sperm were also found in fertilized egg cells [19], [20], indicating the possible involvement of sperm-delivered paternal transcripts in zygote development. Meyer and Scholten (2007) reported that paternal transcripts of 24 genes were found in zygote of maize [14]. Their findings suggest that at least some plant species have evolved a strategy to activate the paternal genome immediately after fertilization. Recently, interleukin-1 receptor–associated kinase (IRAK)/Pelle-like kinase gene SHORT SUSPENSOR (SSP) transcripts, were found to be produced in mature pollen and zygote, where they are translated [13]. Together these data suggest that both sperm-delivered and *de novo* paternal transcripts could be involved in early embryogenesis. However, it is still unknown if paternal transcripts, both sperm delivered and de novo transcribed, exists in interspecies hybrid zygotes, which will indicate if the hybrid vigor can potentially occur from zygote stage. Our present work revealed that among 8 genes expressed in hybrid zygotes, 4 genes were of paternal origin, 1 gene was of maternal origin, and 3 genes were of biparental origin. This suggests the involvement of biparental transcripts in hybrid zygote development of tobacco. Interestingly, some of paternal transcripts were also found in sperm cells, whereas some were not, indicating that the transcripts may come from both sperm delivery during fertilization and *de novo* transcription in zygote.

In wide hybridizations, the parental genome behavior during early embryogenesis has remained unknown. If early embryogenesis is maternally controlled like in animals or some plant species and thus could account for the successful early embryo development and aborted later embryo in hybrid, is a critical question for plant breeders to evaluate parent-fit ability and hybrid vigor. A just published work has reported the dynamic expression of parental-origin genes at early stages of hybrid embryos crossed from different ectypes in *Arabidopsis* [21]. They confirmed again that both paternal and maternal origin genes are involved in early embryogenesis. Our work in tobacco consists with this conclusion. However, based our data we cannot find that dominance of maternal transcripts exist at this stage. Furthermore, our work offers the first evidence for paternal transcript to be involved in zygote development of interspecies hybrids with two possible pathways, as *de novo* transcription in zygote and sperm delivery during fertilization.

### Differential contribution of parental-origin genes in early embryogenesis

Meyer and Scholten (2007) analyzed the allele-specific expression of 25 genes after fertilization of the egg in maize [14]. They used a hybrid crossed between two inbred lines of maize and showed equivalent parental genomic contribution to the hybrid zygote development. This is the first evidence showing comparative parental-origin specific gene expression in a hybrid. In present work we used a hybrid between two different species. We have confirmed the allele-specific expression of 8 genes in tobacco zygotes. The results showed obvious differential contribution of parental-origin genes to the zygote development (Fig 2E). The results indicate that during zygote development and early embryogenesis some of developmental events might be paternally or maternally controlled, while some other processes might be biparentally controlled. This may represent the characteristics of parental-origin gene expression in interspecies hybrid zygotes, as an example of wide hybridization, which is different from the hybrid crossed from two inbred lines of a same species [14].

In addition, we also detected the parent specific expression pattern of the genes at the 8-celled embryo stage. We found that parental-origin gene expression showed different pattern in zygote and 8-celled embryos. Some of the exclusively paternally expressed genes in zygote, e.g. EB426694 and CN744644 showed activation of the maternal allele in 8-celled embryos. These data indicate that the expression pattern of allele specific genes during early embryogenesis is developmental stage-dependent and thus parental and maternal transcripts may have different contribution to the hybrid embryo development at certain stages.

The function of all these parental-origin genes found in this work remains unknown and if they could contribute to the embryo development still needs to be confirmed. Even so, an interspecies hybrid embryo is considered as a convenient model for the study of parental gene expression and the role of the genes in early embryogenesis, especially when the embryos between paternal and maternal parents are morphologically different as what we observed in this work. Presumably, morphological and physiological characters of a hybrid embryo may offer unique clues to distinguish possible heterosis and specific processes of embryogenesis predominantly regulated by parental-origin genes. For this purpose, hybrid embryo development needs to be further investigated.

## Materials and Methods

### Plant material and Growth Conditions

*Nicotiana tabacum* var. SR1 and *N. rustica* var. Hamayan were grown in a greenhouse of Wuhan University at 25°C with a 16/8 h light period.

### Ovule culture

The seedlings of the hybrid (Hamayan × SR1) were obtained by ovule culture in vitro according to established method [15]; the ovules were isolated from ovaries 5–7 day after pollination (DAP) for the culture.

### Interspecies hybridization

For Hamayan × SR1, mature SR1 pollen was directly pollinated on the stigma of Hamayan, which was castrated previously. For SR1 × Hamayan, mature SR1 ovaries were first cut to remove style and treated with sterile pollen culture medium(20℅sucrose, 0.01℅H3BO_3_, 0.1 mM CaCl, pH 5.6) several times. Then, Hamayan pollen was put on the cutting end of SR1 ovaries. Expanded hybrid ovules were isolated from the ovaries at 3–4DAP for observation.

### Observation of the growth of Pollen tube

The method of aniline blue staining was used in this experiment. Pollinated styles was cut open right down the middle before staining, then they were submerged in FAA solution at 4°C overnight. Secondly, styles were put into 5 M NaOH to be soften at room temperature overnight. By cleaned in water a few times, styles were submerged in staining solution (0.1% aniline blue in 10% glycerin) in 1 h, then observed in UV.

### Isolation of sperm cells, zygotes and 8-cell embryos

Sperm cells were isolated as described [16]. For the zygote isolation, the ovaries were cut at 96HAP (day after pollination) from SR1 plants, 35HAP from Hamayan and 48HAP from the hybrid. 8-cell embryos were isolated from SR1 ovaries at 132HAP, Hamayan ovaries at 72 HAP, and hybrid ovaries at 96 HAP, respectively. The method for embryo isolation has been described [17]–[19]. Single cells were transferred to 2×lysis/binding buffer in 0.2 ml tubes and frozen immediately in −80°C refrigerator for later use.

### Construction of cDNA pool

mRNA of Sperm cell was isolated from SR1 by using a Dynabeads mRNA DIRECT Micro Kit (Dynal Biotech). mRNA of zygote and 8-celled embryo was isolated from parents and the hybrid (Hamayan×SR1 ) by using a Dynabeads mRNA DIRECT Micro Kit (Dynal Biotech) A Super SMART™ cDNA PCR Synthesis Kit (Clontech Laboratories) was used for cDNA amplification.

### SSR loci search

ESTs data of tobacco were obtained in FASTA format from NCBI (http://www.ncbi.nlm.nih.gov/). Loci were checked by the MISA (MIcroSAtellites identification tool) for the identification and location of the SSR sites. (http://pgrc.ipk-gatersleben.de/misa/). A DNA sequence assembly program CAP3 was used for the elimination of redundant sequences.

### EST polymorphism detection between SR1 and Hamayan

The primer pairs flanking the SSR loci were designed by using the software primer3 from http://redb.ncpgr.cn/modules/redbtools/primer3.php and synthesized for detecting the polymorphism between SR1 and Hamayan (Table S2). The PCR reaction system was: H_2_O, 0.2–0.4 µl cDNA, 0.2 µM of each primer, 0.2 mM dNTP, 10×R taq reaction buffer (takara) and 0.2 µl R taq DNA polymerase (takara) in a volume of 20 µl. The PCR conditions were 94°C for 30 s, then 35 cycles at 94°C for 30 s, 51–54°C for 30 s, and 72°C for 1 min. The PCR products were detected on 1.5% agarose gels and then the products amplified by the same primers were compared among reactions from SR1, hybrid and Hamayan by PAGE (SDS polyacrylamide gel electrophoresis) for EST polymorphism in 8 % non-denatured gel at 150 V, 20 h.

### Microscopy and image collection

All embryos, ovaries and ovules were observed under an inverted microscope (Olympus IMT-2) and stereoscopic microscope (Olympus SZX12). The images were collected using a cooled charge-coupled device camera (Cool SNAP HQ cooled CCD). Pollen tubes were observed under an inverted microscope (Leica DM IRE2), and images were collected using a cooled CCD camera (MicroMax cooled CCD). The gel images were collected by a scanner (MICROTEK MRS-1200T48U). Photoshop 6.0 and Corel Draw 10.0 were used for image processing.

## Supporting Information

Figure S1 **The time courses of early embryogenesis in SR1, Hamayan and the hybrid (Hamayan × SR1).**
(DOC)

Figure S2 **The Development States of Ovary and Ovule.**
(DOC)

Figure S3 **The Mature Style in SR1 and Hamayan.**
(DOC)

Figure S4 **The growth of Hamayan and SR1 pollen tube in SR1 style.**
(DOC)

Figure S5 **The 2-celled proembryo from the hybrid (SR1 × Hamayan).**
(DOC)

Table S1 **Statistics of hybrid embryos rescued (SR1 × Hamayan).**
(DOC)

Table S2 **Sequences, annealing temperatures and the length of amplified fragments of primers.**
(DOC)
