# Supplementary figures and images for: Genes of Both Parental Origins Are Differentially Involved in Early Embryogenesis of a Tobacco Interspecies Hybrid
Source: PLoS One. 2011 Aug 4;6(8):e23153. doi: 10.1371/journal.pone.0023153 (PMC3150392; doi:10.1371/journal.pone.0023153)

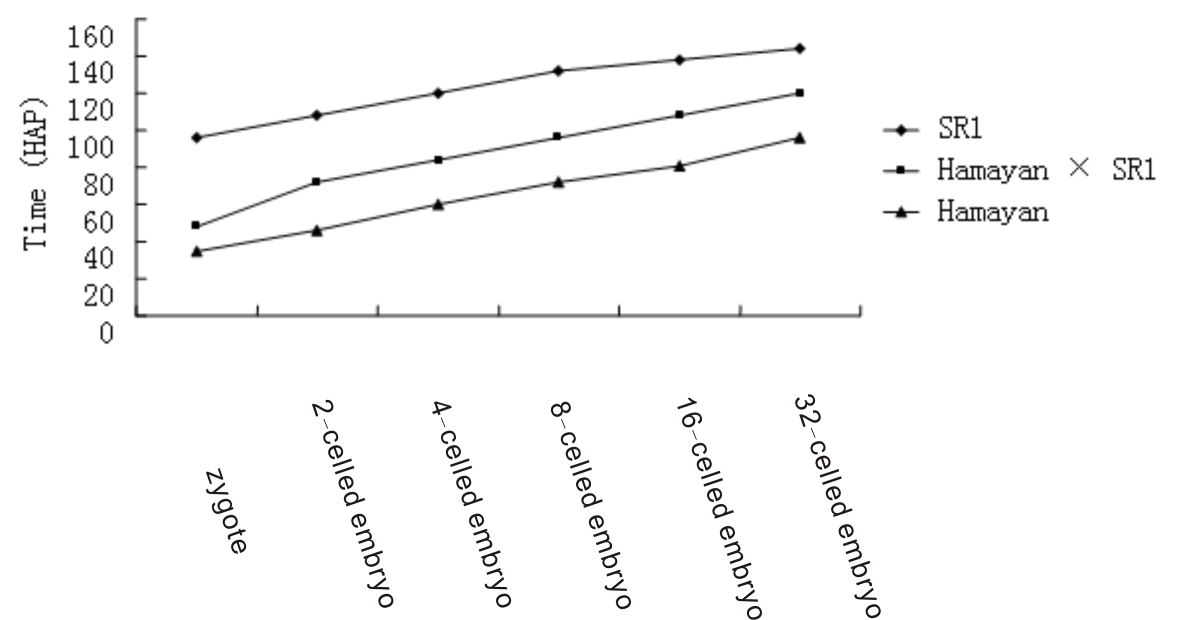


**Figure S1. The time courses of early embryogenesis in SR1, Hamayan and the hybrid (Hamayan × SR1).**

Supplement: Figure S1 — The time courses of early embryogenesis in SR1, Hamayan and the hybrid (Hamayan × SR1). (DOC) [file pone.0023153.s001.doc]

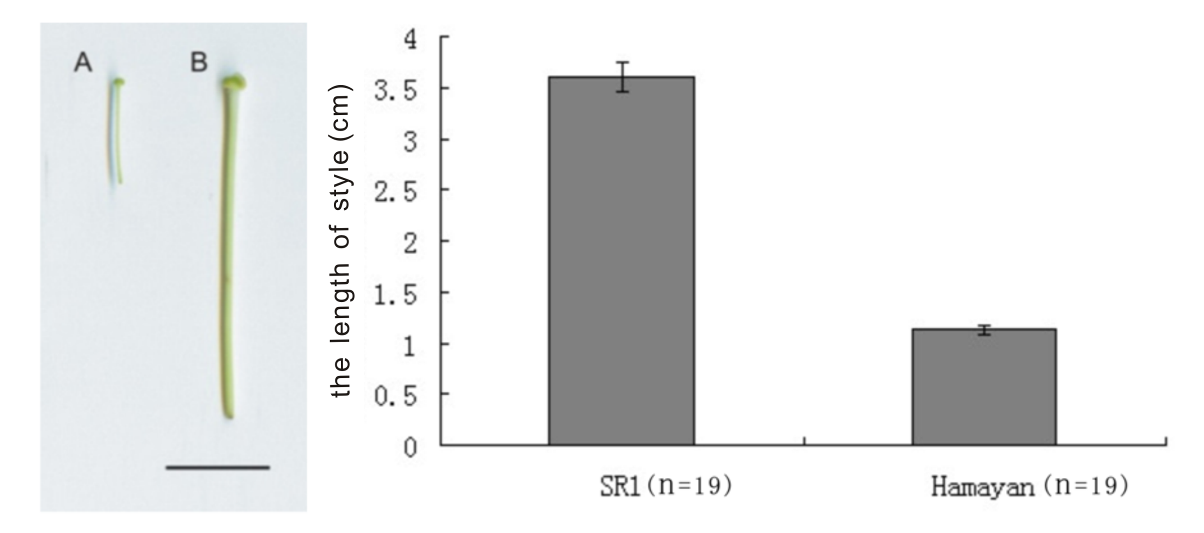


**Figure S3. The Mature Style in SR1 and Hamayan.** (A). The style of Hamayan; (B). The style of SR1. Bar=1cm.

Supplement: Figure S3 — The Mature Style in SR1 and Hamayan. (DOC) [file pone.0023153.s003.doc]

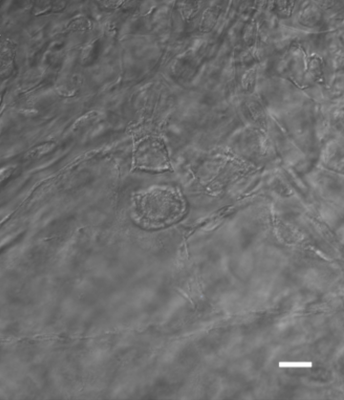


**Figure S5. The 2-celled proembryo from the hybrid (SR1 × Hamayan).** Bar=10μm.

Supplement: Figure S5 — The 2-celled proembryo from the hybrid (SR1 × Hamayan). (DOC) [file pone.0023153.s005.doc]
