# Supplementary material for: Genes of Both Parental Origins Are Differentially Involved in Early Embryogenesis of a Tobacco Interspecies Hybrid
Source: PLoS One. 2011 Aug 4;6(8):e23153. doi: 10.1371/journal.pone.0023153 (PMC3150392; doi:10.1371/journal.pone.0023153)
Supplement: Figure S2 — The Development States of Ovary and Ovule. (DOC) [file pone.0023153.s002.doc]

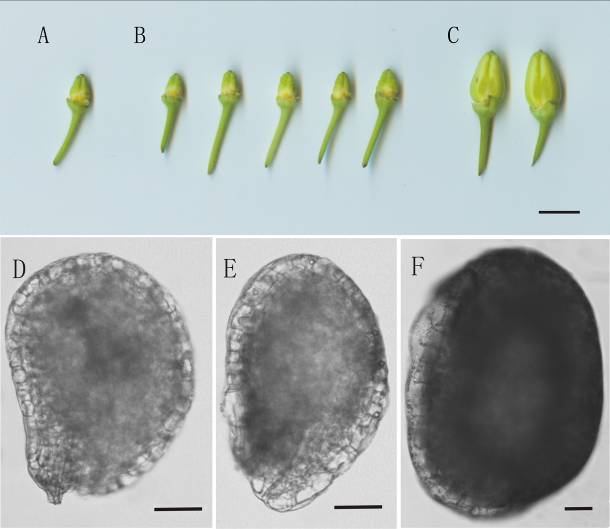


**Figure S2. the Development States of Ovary and Ovule.** (A). Ovary of SR1 without pollination at 96 HAP; (B). Ovary of hybrid (SR1 × Hamayan); (C). Ovary of SR1 was self-pollinated at 96 HAP. Bar=1cm; (D). Ovule of SR1 without pollination at 96 HAP; (E). Ovule of hybrid (SR1 × Hamayan); (F). Ovule of SR1 with self-pollination at 96 HAP. Bar=100μm.
