# Supplementary material for: Genes of Both Parental Origins Are Differentially Involved in Early Embryogenesis of a Tobacco Interspecies Hybrid
Source: PLoS One. 2011 Aug 4;6(8):e23153. doi: 10.1371/journal.pone.0023153 (PMC3150392; doi:10.1371/journal.pone.0023153)
Supplement: Figure S4 — The growth of Hamayan and SR1 pollen tube in SR1 style. (DOC) [file pone.0023153.s004.doc]

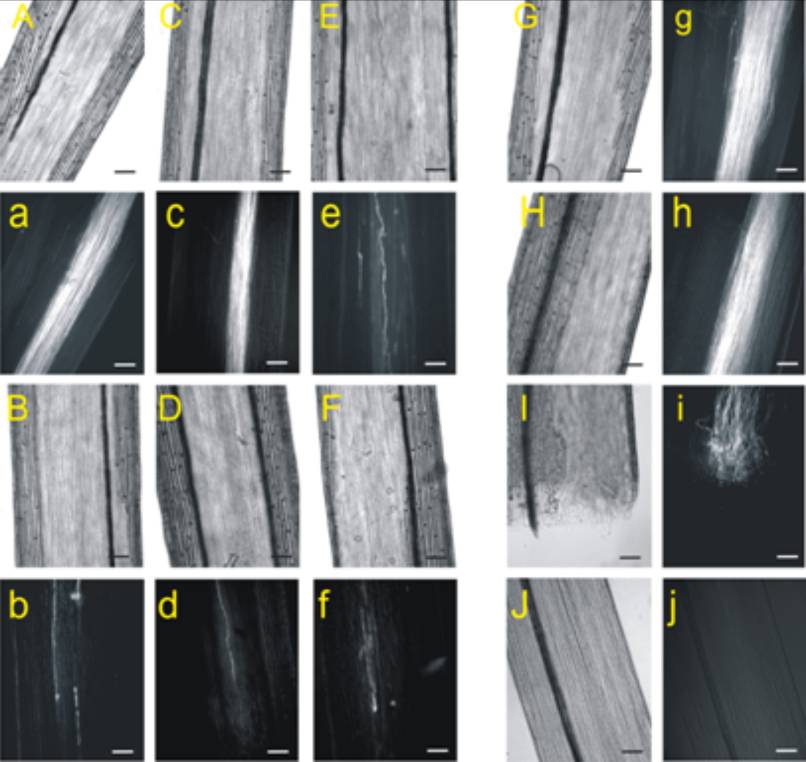


**Figure S4. The growth of Hamayan and SR1 pollen tube in SR1 style.**

(A and a). The growth of Hamayan pollen tube in 1/3 SR1 style at 24 HAP.

(C and c). The growth of Hamayan pollen tube in 1/3 SR1 style at 48 HAP.

(E and e). The growth of Hamayan pollen tube in 1/3 SR1 style at 72 HAP.

(B and b). The growth of Hamayan pollen tube in 2/3 SR1 style at 24 HAP.

(D and d). The growth of Hamayan pollen tube in 2/3 SR1 style at 48 HAP.

(F and f). The growth of Hamayan pollen tube in 2/3 SR1 style at 72 HAP.

(G and g). The growth of SR1 pollen tube in 1/3 SR1 style at 44 HAP.

(H and h). The growth of SR1 pollen tube in 2/3 SR1 style at 44 HAP.

(I and i). The growth of SR1 pollen tube in 3/3 SR1 style at 44 HAP.

(J and j). No pollen tube in SR1 style;

The capitals : bright field images.

The lowercases: fluorescent images. Bar=100μm.
