# Supplementary material for: Genes of Both Parental Origins Are Differentially Involved in Early Embryogenesis of a Tobacco Interspecies Hybrid
Source: PLoS One. 2011 Aug 4;6(8):e23153. doi: 10.1371/journal.pone.0023153 (PMC3150392; doi:10.1371/journal.pone.0023153)
Supplement: Table S1 — Statistics of hybrid embryos rescued (SR1 × Hamayan). (DOC) [file pone.0023153.s006.doc]

**Table S1. Statistics of hybrid embryos rescued (SR1×Hamayan)**

| **Manner of pollination** | **Unpollinated ovaries** | **Pollinated ovaries** | **Fertilized ovaries** | **Total expanded ovules** |
| --- | --- | --- | --- | --- |
| SR1×SR1 | 9 | 9 | 6 | 233 |
| SR1×Hamayan | 9 | 9 | 7 | 60 |
| negative control^a^ | 9 | 0 | 0 | 0 |

^a^ negative control using SR1 ovaries
