# Supplementary material for: Genes of Both Parental Origins Are Differentially Involved in Early Embryogenesis of a Tobacco Interspecies Hybrid
Source: PLoS One. 2011 Aug 4;6(8):e23153. doi: 10.1371/journal.pone.0023153 (PMC3150392; doi:10.1371/journal.pone.0023153)
Supplement: Table S2 — Sequences, annealing temperatures and the length of amplified fragments of primers. (DOC) [file pone.0023153.s007.doc]

**Table S2. Sequences, annealing temperatures and the length of amplified fragments of primers**

| **Accession** | **Amplified fragment length (bp)** | **Repeat units** | **Primers sequences 5/-3/** | **T_a_(℃)** |
| --- | --- | --- | --- | --- |
| DW003872 | 193 | (GAA)_6_ | F: AATCAATTCCAGGCTCATCG | 50 |
|  |  |  | R:TTTTTCCCCTCTTATGCCACT |  |
| EB698387 | 200 | (AGT)_7_ | F: TGGTGGTCAAAGAGGGAATC | 52 |
|  |  |  | R: TGATCCCTGAAGGAAAATGG |  |
| BP135503 | 200 | (CCT)_7_ | F: GCACATGTGGTGGTTACAGC | 54 |
|  |  |  | R: CGGTCGTGGTTGTGTTTATG |  |
| EB426694 | 189 | (GAG)_7_ | F: GGT GAT CCA TTC GGT TAT CG  R: CCG ACA ACC TCA ACA ATC CT | 53 |
| DV999024 | 199 | (TAG)_7_ | F: AAGCAGCGGAAAAAT ATG GA  R: AGCACTGAAAATCCG AGT CAA | 50 |
| DV998760 | 201 | (TCT)_8_ | F: CTG AAA GGG CAT GGA AGT GT  R: TAG CAA CCG TCT TCA GCA AA | 52 |
| CN744644 | 195 | (GAG)_9_ | F: GGT GAT CCA TTC GGT TAT CG  R: CCG ACA ACC TCA ACA ATC CT | 53 |
| CN747982 | 215 | (TTCATC)_5_ (CTT)_6_ | F: TGC TCC TCC ACT CCT CTT GT  R: GGG AAG CTG CTC AGG ATA AA | 54 |
